# Selections

**Published:** 1892-10

**Authors:** 


### Selections.

### EUROPHEN IN MINOR SURGERY.

THE results obtained by me from the use of europhen have been most satisfactory, and lead me to add my indorsement of its use as a substitute for iodoform.

In the case of a driver of a beer-wagon, the index and middle fingers of the right hand had been severely crushed by being caught between two barrels. There was a fracture of the middle phalanx of the index finger, with several deep, lacerated wounds of both fingers. Several days before coming to me the patient had applied a carbolic-acid solution to the fingers, which had become of a greenish-black color. The fingers and hand were intensely swollen; there was pain in the elbow, and a dark, foul-smelling pus exuded from the wounds. The treatment was by thorough washing of the injured parts in bichloride solution, insufflation of europhen into the wounds, and envelopment of the fingers in a one-to-eight ointment of europhen and lanolin. Five days therefrom the swelling of the fingers had become reduced and there was less pus secretion from the wounds. The skin was still black and easily detached from the fingers in several places. A dark pus covered the denuded surfaces. As much of the cuticle as could be separated was removed, and europhen was dusted upon the denuded surfaces, as also into the wounds. This treatment was continued, and in about eighteen days the wounds were entirely closed, the cuticle having been freely and completely separated two days previously.

A child, four years old, was suffering from a large cervical abscess. It was opened and a large quantity of pus evacuated. The abscess cavity was curetted and europhen insufflated. The child was seen several days thereafter. Recovery was uninterrupted.

A child, aged three years, had had its buttocks and left lower extremity severely scalded by falling into a pot of boiling water. Carron oil was applied for forty-eight hours, followed by the dusting of europhen over the buttocks and half of the thigh. Boric acid and bismuth were used on the rest of the thigh and on the leg. As no deleterious results followed the application of europhen, its use was adopted upon the whole of the scalded surface. Recovery was complete with no untoward symptoms.

In several other minor cases I have used europhen, and in no case have poisonous results followed its application. Europhen is undoubtedly as effective as iodoform. It is lighter in weight, does not cake, and is more readily dusted or insufflated. It is innocuous and free from disagreeable odor.

P. G. Becker, M.D.

—*New York Medical Journal.*

